# Antimicrobial resistance surveillance and focused comparative genomics of clinical *Salmonella* isolates from patients with diarrhea in northern Gyeonggi Province, Korea

**DOI:** 10.1128/spectrum.03400-25

**Published:** 2026-07-14

**Authors:** Bu-Geon Lim, Kyung-A Kim, Mi-Jung Jang, Hee-Cheon Moon, Myung-Jin Lee, Bo-Yeon Kweon, Ga-Yeon Kim

**Affiliations:** 1 Northern Branch, Gyeonggi Province Institute of Health and Environment, Uijeongbu, Korea; 2 Gyeonggi Province Institute of Health and Environment 591859 , Suwon, Korea; 3 Department of Public Health, Graduate School, Dankook University 65383, Cheonan, Korea; Ruhr-Universitat Bochum, Bochum, Germany

**Keywords:** WGS, *Salmonella enterica*, PMQR, PFGE, MLST, ESBL

## Abstract

We analyzed 320 *Salmonella enterica* isolates recovered from patients with diarrhea in northern Gyeonggi Province, Korea, between 2015 and 2022. Resistance was most frequent to ampicillin, tetracycline, and nalidixic acid, and 26.6% of isolates exhibited multidrug resistance (MDR). Extended-spectrum cephalosporin-resistant isolates mainly carried *bla*_CTX-M_ and *bla*_DHA_, whereas plasmid-mediated quinolone resistance determinants, such as *qnrS* and *qnrB*, were also detected. Mutations in quinolone resistance-determining regions, predominantly *gyrA* mutations corresponding to the predicted D87N substitution, were frequent. PFGE and MLST supported relatedness within major resistant serotypes; ST34 and ST11 were the most frequent sequence types among the MLST-analyzed isolates. Resistance to nalidixic acid and colistin was significantly higher in *S*. Enteritidis than in *S*. I 4,[5],12:i:-, whereas *S*. I 4,[5],12:i:- showed higher resistance to tetracycline, chloramphenicol, and trimethoprim-sulfamethoxazole (all *P* < 0.001). Whole-genome sequencing of two representative isolates (GGN-BG-22, ST34; GGN-BG-25, ST11) identified distinct resistance- and virulence-related genomic features, including plasmid-associated resistance determinants. SNP-based phylogenetic analyses using selected publicly available genomes placed these two representative lineages in a broader genomic context. Together, these findings provide regional surveillance-based insights into antimicrobial resistance in clinical *Salmonella* isolates and focused genomic insights into two clinically relevant lineages.

**IMPORTANCE:**

Multidrug-resistant *Salmonella* poses an increasing challenge to both patient care and public health. This study integrates regional antimicrobial resistance surveillance with focused comparative genomic analysis of two representative clinical isolates, providing context for major resistant lineages identified in northern Gyeonggi Province, Korea. Our findings highlight lineage-specific resistance and genomic features and support the continued use of molecular surveillance to monitor high-risk clones and resistance-associated mobile genetic elements in clinical and public health settings.

## INTRODUCTION

*Salmonella enterica* remains a major foodborne pathogen causing substantial morbidity worldwide and is commonly associated with diarrheal illness in humans (1). The increasing emergence of multidrug-resistant (MDR) *Salmonella*, particularly isolates resistant to extended-spectrum cephalosporins and fluoroquinolones, has become an important challenge for both clinical treatment and public health control (2–4). The dissemination of extended-spectrum β-lactamase genes and plasmid-mediated quinolone resistance (PMQR) determinants, along with the accumulation of mutations in quinolone resistance-determining regions (QRDRs), has further limited available treatment options (5, 6).

Molecular subtyping methods, such as pulsed-field gel electrophoresis (PFGE) and multilocus sequence typing (MLST), have been widely used to assess clonal relatedness and track the dissemination of resistant *Salmonella* lineages (7–9). More recently, whole-genome sequencing (WGS) has enabled higher-resolution characterization of antimicrobial resistance determinants, mobile genetic elements, and virulence-associated loci (10, 11). Used together, these approaches can provide complementary information on the population structure and genomic features of clinically important lineages.

In Korea, previous studies have described the serotype distribution and antimicrobial resistance patterns of *Salmonella* isolates, and recent reports have increasingly incorporated WGS-based analyses (12–14). Building on these approaches, we investigated *Salmonella enterica* isolates recovered from patients with diarrhea in northern Gyeonggi Province, Korea, between 2015 and 2022.

The aims of this study were to determine the serotype distribution and antimicrobial resistance profiles of the isolates, identify β-lactamase genes and PMQR determinants together with QRDR mutations, and assess clonal relationships using PFGE and MLST.

In addition, WGS of two representative MDR isolates was performed to provide focused comparative genomic context for major resistant lineages identified in this regional clinical collection.

## MATERIALS AND METHODS

### Bacterial isolates

Between January 2015 and December 2022, 320 *Salmonella* isolates were obtained from rectal swab specimens of patients with diarrhea in northern Gyeonggi Province, Korea. Specimens were collected by local public health centers during outbreak investigations and submitted to the Gyeonggi Province Institute of Health and Environment under the Infectious Disease Control and Prevention Act. The specimens were used solely for diagnostic purposes, and all identifiers and residual materials were discarded after pathogen confirmation. Only the isolates were retained in a de-identified form for analysis.

### Isolation and culture

Rectal swab specimens were inoculated into 9 mL of tryptic soy broth (Difco Laboratories, Detroit, MI, USA) and incubated at 37°C for 18 h. Aliquots were streaked onto *Salmonella*-*Shigella* (SS) agar (Difco Laboratories) and cultured at 37°C for 18 h. Presumptive colonies were purified, and confirmation of *Salmonella*-specific genes was performed using a PowerChek Gram-Negative Multiplex Detection PCR kit (Kogene Biotech, Seoul, Korea). Biochemical identification was conducted using a VITEK 2 COMPACT system (BioMérieux, Lyon, France).

Isolates were serotyped and preserved in CryoCare Bacterial Preservation vials (Key Scientific Products, Stamford, TX, USA) at −75°C until further use.

### Serotyping

Serotyping was performed according to the Kauffmann–White classification scheme using O antigen antisera and H antigen antisera (Difco Laboratories) (15). O antigens were determined using slide agglutination, while H antigens (phases 1 and 2) were identified using motility GI medium and veal infusion broth, followed by formalin fixation and agglutination testing with specific antisera. Final serotypes were assigned based on combinations of O and H antigen patterns.

### Antimicrobial susceptibility testing

Antimicrobial susceptibility was determined for all 320 isolates using a Sensititre KRCDC2F custom plate (Thermo Fisher Scientific, Waltham, MA, USA) following the Clinical and Laboratory Standards Institute (CLSI) guidelines (M100, 35th ed., 2025) (16). Minimum inhibitory concentrations (MICs) were determined using broth microdilution. Bacteria were grown on tryptic soy agar (Difco Laboratories) for 18 h at 37°C, suspended to 0.5 McFarland turbidity, diluted in cation-adjusted Mueller–Hinton broth (CA-MHB; TREK), and inoculated into microtiter wells. Plates were incubated at 36°C for 20 h, and MICs were recorded as the lowest concentration inhibiting visible growth. For quality control, *Escherichia coli* ATCC 25922 was included. The antibiotics tested included ciprofloxacin (CIP), imipenem (IPM), ampicillin (AMP), tetracycline (TET), gentamicin (GEN), nalidixic acid (NAL), cefoxitin (FOX), colistin (COL), cefotaxime (CTX), ceftriaxone (CRO), ceftazidime (CAZ), chloramphenicol (CHL), amikacin (AMK), and trimethoprim-sulfamethoxazole (SXT).

### Detection of AMR genes

Isolates resistant to third-generation cephalosporins (*n* = 24) were screened for *bla*_CTX-M_, *bla*_TEM_, *bla*_OXA_, *bla*_SHV_, *bla*_CMY_, and *bla*_DHA_ genes using PCR (17). Genomic DNA was extracted using a QIAamp DNA Mini Kit (Qiagen, Hilden, Germany), and PCR amplification was performed using an AccuPower Gold Multiplex PCR PreMix Kit (Bioneer, Daejeon, Korea).

Products were analyzed using gel electrophoresis, and representative amplicons were sequenced (Macrogen, Seoul, Korea). Sequences were analyzed using NCBI BLAST for allele identification. Among the isolates, 110 with resistance or intermediate resistance to NAL or CIP were analyzed for mutations in *gyrA*, *gyrB*, *parC*, and *parE* (QRDRs) (18), as well as for PMQR determinants, including *qnrA*, *qnrB*, *qnrC*, *qnrD*, *qnrS*, *aac(6*′)*-Ib-cr*, *oqxA*, *oqxB*, and *qepA* (19–23). Primer sequences and PCR conditions are provided in Tables S1 and S2.

### Statistical analysis

To assess differences in antimicrobial resistance between *S*. Enteritidis and *S*. I 4,[5],12:i:-, isolates were categorized into resistant and non-resistant groups for each drug, with intermediate and susceptible categories combined as non-resistant where applicable. The resulting 2 × 2 contingency tables were analyzed using Fisher’s exact test, with statistical significance defined as a *P* value < 0.05. All analyses were conducted in R software (version 4.3.2; R Foundation for Statistical Computing, Vienna, Austria).

### PFGE analysis

PFGE was performed according to the KCDA protocol (2022) (24) to evaluate genetic relatedness. Briefly, bacterial cells were immobilized in agarose plugs, lysed with proteinase K in ES buffer, and subsequently treated with XbaI (Roche Diagnostics, Rotkreuz, Switzerland) for 6 h at 37°C. The resulting DNA fragments were separated on a CHEF-DR III System (Bio-Rad Laboratories, Hercules, USA) under the following parameters: switch times from 2.16 to 63.8 s, 6 V cm⁻^1^, 14°C, for 18 h. Gels were stained with SYBR Gold (Invitrogen, Carlsbad, CA, USA), destained, and visualized using UV illumination. *S*. Braenderup (ATCC BAA-664) served as the molecular size standard, and banding patterns were analyzed using BioNumerics (BioMérieux).

### MLST analysis

MLST followed the *Salmonella enterica* scheme based on seven housekeeping loci (*aroC*, *dnaN*, *hemD*, *hisD*, *purE*, *sucA*, and *thrA*). PCR amplicons were sequenced by Macrogen Inc. (Seoul, Korea), and allele numbers and sequence types (STs) were assigned using the PubMLST *Salmonella* database (25). Two alleles (*hisD* and *thrA*) that did not match existing entries were identified as novel alleles; however, official numbers were not assigned under the updated PubMLST policy requiring WGS submission. The concatenated sequences were subjected to MUSCLE alignment, and phylogenies were reconstructed in MEGA version 12 using the neighbor-joining approach with the Tamura–Nei model and 1,000 bootstrap replicates. Only bootstrap values of 70% or above were considered to indicate robust support.

### Whole-genome sequencing and *in silico* analyses

WGS was applied to two MDR isolates (GGN-BG-22 and GGN-BG-25), which were selected as representative isolates based on their resistance phenotypes and epidemiological significance. *Salmonella enterica* serovar I 4,[5],12:i:- is the monophasic variant of *S*. Typhimurium. In this study, GGN-BG-22 was identified as an ST34 isolate of this serovar, whereas GGN-BG-25 was an ST11 *S*. Enteritidis isolate. These isolates were sequenced using the PacBio Sequel II and Illumina NovaSeq 6000 platforms by Macrogen. Assembled genome sequences were submitted to GenBank with the accession numbers CP196701 (chromosome) and CP196702 (plasmid 1) for GGN-BG-22 and CP196402 (chromosome) and CP196403 (plasmid 1) for GGN-BG-25.

Genomic characterization, including MLST, cgMLST, wgMLST, rMLST, and *in silico* serotyping, was performed using EnteroBase (26). Genome annotation was conducted using the Bakta annotation tool implemented in Proksee (27, 28), with database version 5.0, incorporating CARD RGI 6.0.3, ResFinder 4.7.2, MobileElementFinder 1.0.3, MobileOG-db beatrix-1.6, and VirulenceFinder 2.0 resources (29–33). Plasmid replicon typing was performed using PlasmidFinder 2.1 (34), and plasmid maps were visualized and annotated using Proksee. To assess the genomic context of chromosomal *sopE*, prophage-associated regions were additionally analyzed using PHASTEST (35). Single-nucleotide polymorphism (SNP)-based phylogenetic trees were generated using CSI Phylogeny 1.4 (Center for Genomic Epidemiology, Kongens Lyngby, Denmark), with USDA15WA-1 (CP040686) and P125109 (NC_011294) used as reference strains for *S*. I 4,[5],12:i:- and *S*. Enteritidis, respectively (36). Comparator genomes were selected from publicly available GenBank records for the corresponding sequence types that were available at the time of analysis. The comparator genomes and their metadata are listed in Tables S3 and S4.

The phylogenetic trees were visualized using Interactive Tree of Life (iTOL) (37). In total, 445 and 463 high-quality core SNPs were identified for the monophasic variant of *S*. Typhimurium and *S*. Enteritidis data sets, respectively.

## RESULTS

### Serotype distribution of *Salmonella* isolates

As shown in Table 1, *S*. Enteritidis (*n* = 89, 27.8%) and *S*. I 4,[5],12:i:- (*n* = 65, 20.3%) were the most common serotypes, followed by *S*. Bareilly (*n* = 24, 7.5%), *S*. Typhimurium (*n* = 22, 6.9%), *S*. Infantis (*n* = 21, 6.6%), *S*. Thompson (*n* = 18, 5.6%), and *S*. Agona (*n* = 13, 4.1%). The remaining 68 isolates (≤3.0% each) comprised 25 additional serotypes, including *S*. Newport, *S*. Stanley, *S*. Saintpaul, and *S*. Virchow.

**TABLE 1:** Annual distribution of major *Salmonella* serotypes among 320 isolates recovered from patients with diarrhea in northern Gyeonggi Province, Korea, 2015–2022

Year	Enteritidis	I 4,[5],12:i:-	Bareilly	Typhimurium	Infantis	Thompson	Agona	Others^*a*^
2015	0	2	0	0	0	0	0	5
2016	2	4	1	0	4	0	0	8
2017	3	11	4	2	1	4	2	7
2018	0	7	2	1	3	6	1	5
2019	1	6	5	3	1	0	3	10
2020	5	3	0	0	1	1	0	3
2021	53	8	2	3	4	2	0	1
2022	25	24	10	13	7	5	7	29
Total (%)	89 (27.8)	65 (20.3)	24 (7.5)	22 (6.9)	21 (6.6)	18 (5.6)	13 (4.1)	68 (21.2)

^*a*^ Others include 25 additional serotypes, each accounting for <3.0% of the 320 isolates.

### Antimicrobial susceptibility and MDR profiles

Antimicrobial susceptibility testing of the 320 isolates, as shown in Table 2, revealed the highest resistance to AMP (41.3%) and TET (34.4%), followed by NAL (30.6%), COL (10.6%), CHL (10.3%), CTX (7.5%), CRO (7.2%), and CAZ (6.3%). All isolates remained susceptible to IPM and AMK, while >98% were susceptible to FOX and >94% to GEN. In total, 85 isolates (26.6%) were classified as multidrug resistant, defined as resistance to three or more antimicrobial classes. Among MDR isolates, the most frequent MDR profile was NAL–AMP–TET (22.4%); detailed MDR combinations are provided in Table S5.

**TABLE 2:** Antimicrobial susceptibility profiles of 320 *Salmonella* isolates recovered from patients with diarrhea in northern Gyeonggi Province, Korea^*a*^

Antimicrobial agent	Susceptible, *n* (%)	Intermediate, *n* (%)	Resistant, *n* (%)
Ciprofloxacin	297 (92.8)	20 (6.3)	3 (0.9)
Nalidixic acid	222 (69.4)	0 (0.0)	98 (30.6)
Colistin	–	286 (89.4)	34 (10.6)
Imipenem	320 (100.0)	0 (0.0)	0 (0.0)
Cefotaxime	295 (92.2)	1 (0.3)	24 (7.5)
Ceftriaxone	297 (92.8)	0 (0.0)	23 (7.2)
Ceftazidime	299 (93.4)	1 (0.3)	20 (6.3)
Cefoxitin	314 (98.1)	4 (1.3)	2 (0.6)
Ampicillin	188 (58.8)	0 (0.0)	132 (41.3)
Tetracycline	210 (65.6)	0 (0.0)	110 (34.4)
Chloramphenicol	286 (89.4)	1 (0.3)	33 (10.3)
Gentamicin	302 (94.4)	3 (0.9)	15 (4.7)
Amikacin	320 (100.0)	0 (0.0)	0 (0.0)
Trimethoprim-sulfamethoxazole	301 (94.1)	0 (0.0)	19 (5.9)

^*a*^ Values are presented as number (%) of isolates among 320 total isolates. –, not applicable. For colistin, MIC values were interpreted as intermediate (≤2 μg/mL) or resistant (≥4 μg/mL) because no susceptible category is defined for Enterobacterales.

When resistance profiles of *S*. Enteritidis (*n* = 89) and *S*. I 4,[5],12:i:- (*n* = 65) were compared, clear differences emerged (Table 3). Resistance to NAL and COL was substantially higher in *S*. Enteritidis (84.3% and 37.1%) than in *S*. I 4,[5],12:i:- isolates (10.8% and 1.5%; both *P* < 0.001). Conversely, *S*. I 4,[5],12:i:- isolates exhibited greater resistance to TET (73.8% vs 46.1%), CHL (27.7% vs 2.2%), and SXT (20.0% vs 1.1%), with all comparisons reaching statistical significance (*P* < 0.001).

**TABLE 3:** Comparison of antimicrobial resistance between *S*. Enteritidis (*n* = 89) and *S*. I 4,[5],12:i:- (*n* = 65) isolates^*a*^

Antimicrobial agent	*S*. Enteritidis resistant, *n* (%)	*S*. I 4,[5],12:i:- resistant, *n* (%)	*P* value
Ciprofloxacin	0 (0.0)	1 (1.5)	0.422
Nalidixic acid	75 (84.3)	7 (10.8)	6.8 × 10⁻²¹
Colistin	33 (37.1)	1 (1.5)	1.9 × 10⁻⁸
Imipenem	0 (0.0)	0 (0.0)	1.000
Cefotaxime	6 (6.7)	10 (15.4)	0.109
Ceftriaxone	6 (6.7)	9 (13.8)	0.173
Ceftazidime	6 (6.7)	7 (10.8)	0.394
Cefoxitin	0 (0.0)	1 (1.5)	0.422
Ampicillin	59 (66.3)	51 (78.5)	0.108
Tetracycline	41 (46.1)	48 (73.8)	8.8 × 10⁻⁴
Chloramphenicol	2 (2.2)	18 (27.7)	3.2 × 10⁻⁶
Gentamicin	8 (9.0)	2 (3.1)	0.192
Amikacin	0 (0.0)	0 (0.0)	1.000
Trimethoprim-sulfamethoxazole	1 (1.1)	13 (20.0)	5.8 × 10⁻⁵

^*a*^ Values are presented as resistant isolates, *n* (%). *P* values were calculated using Fisher’s exact test comparing resistant and non-resistant isolates. *P* values <0.05 were considered statistically significant.

### β-lactamase genes, QRDR mutations, and PMQR determinants

Among the 24 isolates that were resistant to third-generation cephalosporins, *bla*_CTX-M_ genes were identified in 23, including *bla*_CTX-M-55_ (47.8%), *bla*_CTX-M-15_ (34.8%), *bla*_CTX-M-65_ (13.0%), and *bla*_CTX-M-14_ (4.3%). *bla*_DHA-33_ was detected in a single isolate, whereas *bla*_TEM-1_ was additionally identified in three isolates (Table 4). Among 110 isolates with reduced susceptibility to NAL or CIP, QRDR mutations were observed in 97 (88.2%). The most common QRDR finding involved mutations in *gyrA* corresponding to the predicted D87N substitution, followed by mutations corresponding to the predicted D87Y, D87G, S83Y, and S83F substitutions. Detailed QRDR mutation patterns and PMQR determinants are presented in Tables S6 and S7.

**TABLE 4:** Distribution of β-lactamase genes and subtypes among 24 extended-spectrum cephalosporin-resistant *Salmonella* isolates

β-lactamase gene	Gene subtype	Serotype	No. of isolates
*bla*_CTX-M_ (Group I)	*bla*_CTX-M-15_	*S*. Enteritidis	6
		*S*. Virchow	2
	*bla*_CTX-M-55_	*S*. I 4,[5],12:i:-	7
		*S*. Typhimurium	2
		*S*. Bareilly	1
		*S*. Infantis	1
*bla*_CTX-M_ (Group IV)	*bla*_CTX-M-14_	*S*. I 4,[5],12:i:-	1
	*bla*_CTX-M-65_	*S*. I 4,[5],12:i:-	1
		*S*. Typhimurium	1
		*S*. Thompson	1
*bla*_CTX-M_ (Group II)	Not detected	Not detected	0
*bla*_TEM_	*bla*_TEM-1_	*S*. I 4,[5],12:i:-	2^*a*^
		*S*. Thompson	1^*a*^
*bla*_SHV_	Not detected	Not detected	0
*bla*_OXA_	Not detected	Not detected	0
*bla*_CMY_	Not detected	Not detected	0
*bla*_DHA_	*bla*_DHA-33_	*S*. I 4,[5],12:i:-	1

^*a*^ The *bla*_TEM-1_-positive isolates also carried *bla*_CTX-M_ genes and were therefore counted in both gene categories.

### PFGE and MLST analyses

**Fig 1:**
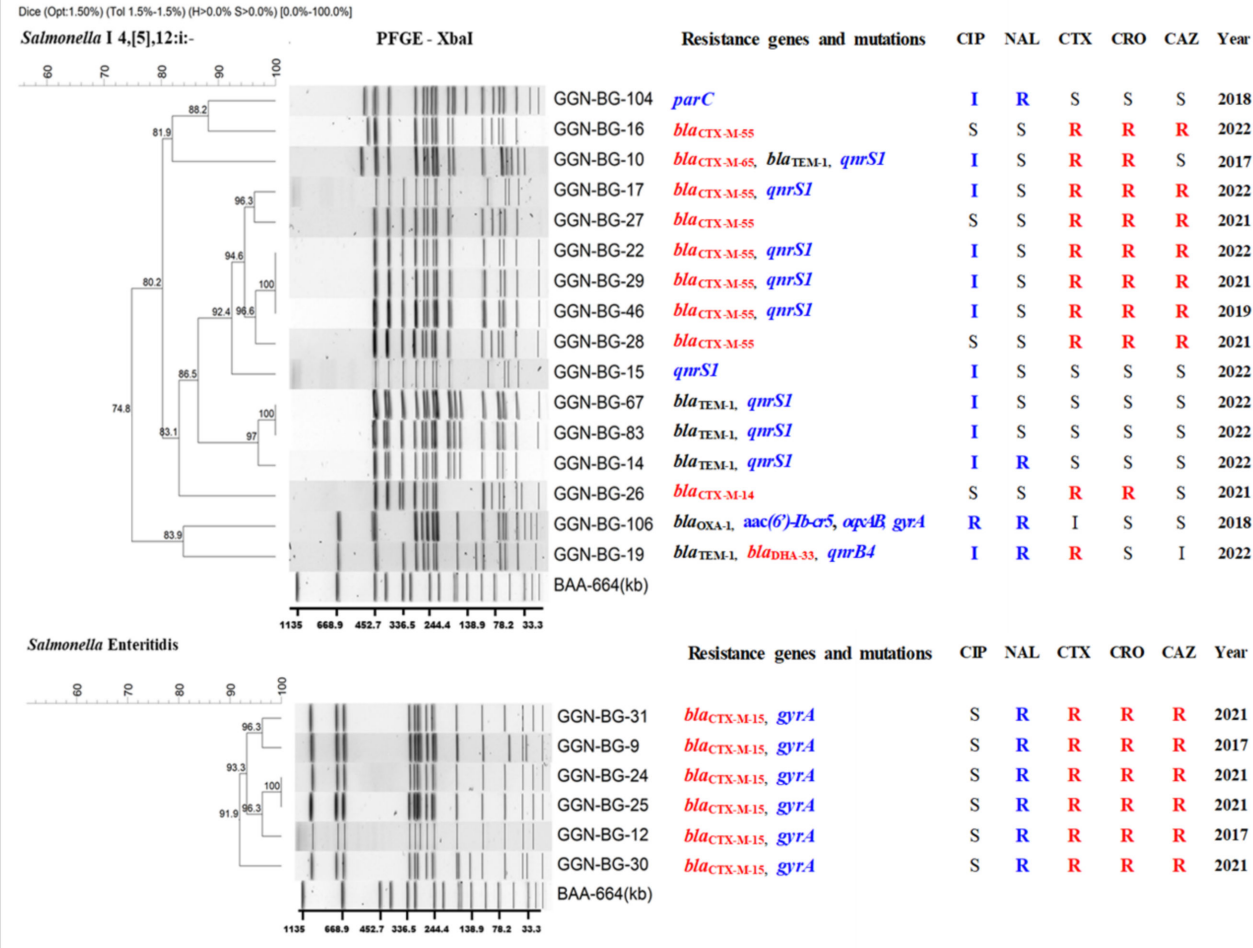
Pulsed-field gel electrophoresis dendrogram of *S*. I 4,[5],12:i:- and *S*. Enteritidis isolates showing resistance to third-generation cephalosporins and/or reduced susceptibility to nalidixic acid or ciprofloxacin. Isolates are clustered by serotype, with accompanying metadata indicating resistance genes, QRDR mutations, antimicrobial susceptibility profiles (R, resistant; I, intermediate; S, susceptible), and year of isolation. Extended-spectrum β-lactamase-related genes and cephalosporin resistance are highlighted in red, whereas quinolone resistance-related genes, QRDR mutations, and quinolone/fluoroquinolone non-susceptibility are shown in blue.

Thirty-five isolates showing resistance to third-generation cephalosporins and/or reduced susceptibility to nalidixic acid or ciprofloxacin were analyzed using PFGE. The major PFGE clusters of *S*. I 4,[5],12:i:- and *S*. Enteritidis are shown in Fig. 1, whereas PFGE patterns of additional serotypes are presented in Fig. S1.

MLST of 38 isolates identified 12 STs. The most common STs were ST34 (39.5%) and ST11 (15.8%), followed by ST203 (13.2%), ST323 (5.3%), and ST16 (5.3%). *S*. I 4,[5],12:i:- isolates were predominantly ST34, whereas *S*. Enteritidis and *S*. Bareilly were assigned to ST11 and ST203, respectively. Detailed ST assignments are provided in Table S8, and an MLST-based phylogenetic tree of 35 selected isolates representing five major serotypes is provided in Fig. S2.

### WGS of representative isolates

WGS was performed on two MDR isolates, *S*. I 4,[5],12:i:- GGN-BG-22 and *S*. Enteritidis GGN-BG-25, using PacBio Sequel II and Illumina platforms (Macrogen). Assembled genome sequences and *in silico* typing results are summarized in Table 5, and raw sequencing reads are available under the corresponding SRA accessions. EnteroBase typing confirmed ST34 (eBG1) and ST11 (eBG4), with cgMLST demonstrating phylogenetic divergence (GGN-BG-22: HC10 359668; GGN-BG-25: HC10 58360).

**TABLE 5:** Genomic features and *in silico* typing results of two representative multidrug-resistant *Salmonella* isolates, GGN-BG-22 and GGN-BG-25^*a*^

Features	GGN-BG-22	GGN-BG-25
Chromosome length (bp)	4,971,900	4,680,720
Plasmid length (bp)	107,357	109,440
Plasmid replicon type	IncFIB(AP001918)	IncFIB(S); IncFII(S)
wgMLST ST	ST741476	ST741475
cgMLST ST	ST636237	ST636236
HC0	636,237	636,236
HC10	359,668	58,360
HC100	2	87
7-gene MLST ST	ST34 (eBG1)	ST11 (eBG4)
7-gene allele profile	10-19-12-9-5-9-2	5-2-3-7-6-6-11
rMLST ST	ST1369 (eBG1)	ST1425 (eBG4.1)
*In silico* serovar (SISTR)	*S*. I 4,[5],12:i:-	*S*. Enteritidis
EnteroBase assembly ID	SAL_KE5983AA_AS	SAL_KE5982AA_AS
GenBank accession	CP196701, CP196702	CP196402, CP196403
SRA accession	SRR34534925; SRR34539401	SRR34534924; SRR34541762
BioProject	PRJNA1290791	PRJNA1290791
BioSample	SAMN49937988	SAMN49937989

^*a*^ ST, sequence type; MLST, multilocus sequence typing; wgMLST, whole-genome MLST; cgMLST, core genome MLST; rMLST, ribosomal MLST; eBG, eBurst Group; HC, hierarchical cluster (e.g., HC0, identical alleles; HC100, ≤100 allelic differences); SISTR, *Salmonella* in silico serotyping resource.

### Chromosomal and plasmid features

**Fig 2:**
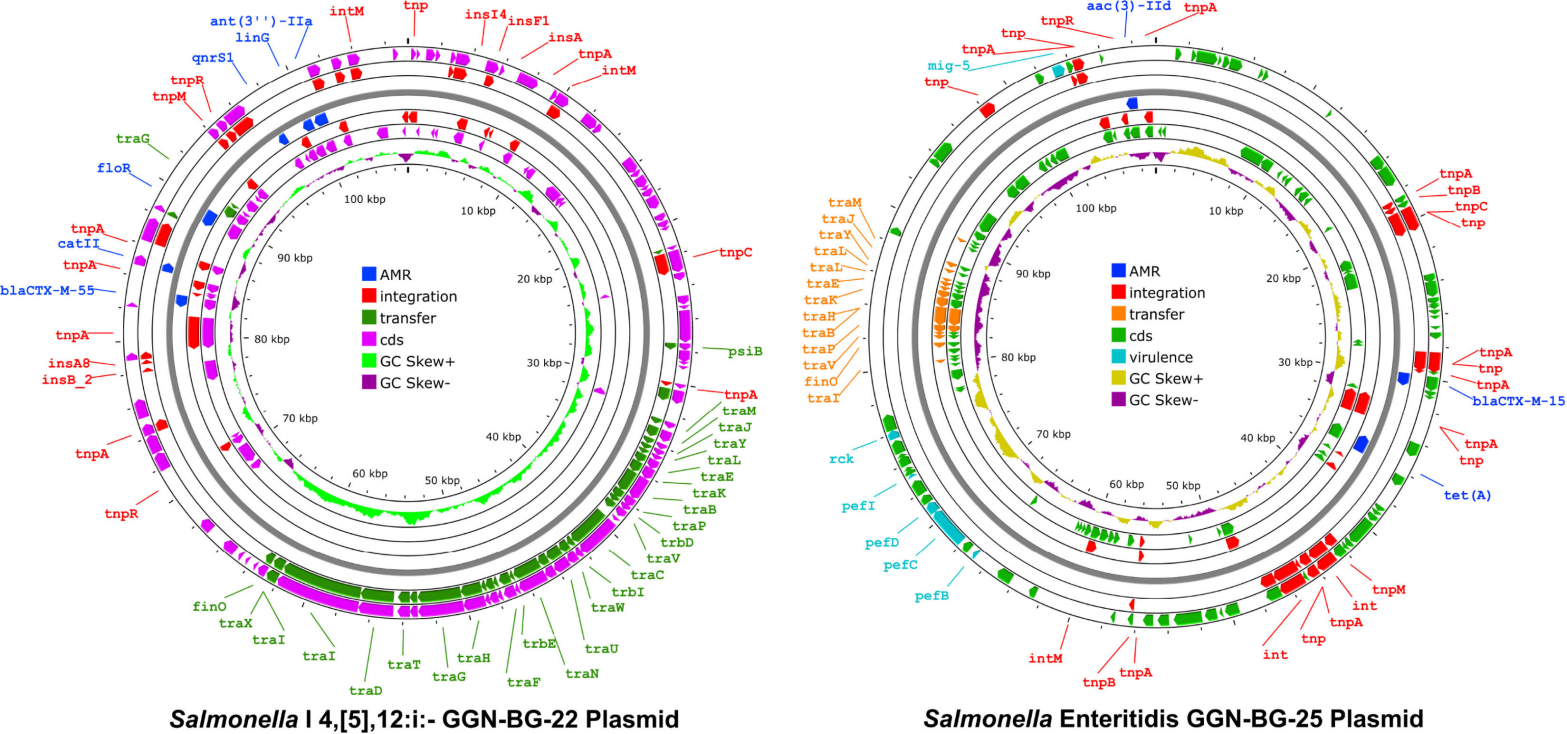
Comparative circular plasmid maps of *S*. I 4,[5],12:i:- isolate GGN-BG-22 and *S*. Enteritidis isolate GGN-BG-25. The maps show coding sequences, antimicrobial resistance (AMR) genes, mobile genetic element-associated genes, conjugative transfer genes, virulence-associated genes, and GC skew. The GGN-BG-22 plasmid carried an IncFIB(AP001918) replicon and harbored *bla*_CTX-M-55_, *qnrS1*, *floR*, *catA2*, and *lnu(F*), together with an *ant(3*″)-*IIa*-like aminoglycoside resistance gene annotated as a pseudogene, whereas the GGN-BG-25 plasmid carried IncFIB(S) and IncFII(S) replicons and harbored *bla*_CTX-M-15_, *tet(A*), and *aac(3)-IId*. AMR regions in both plasmids were associated with mobile genetic element-related genes.

**Fig 3:**
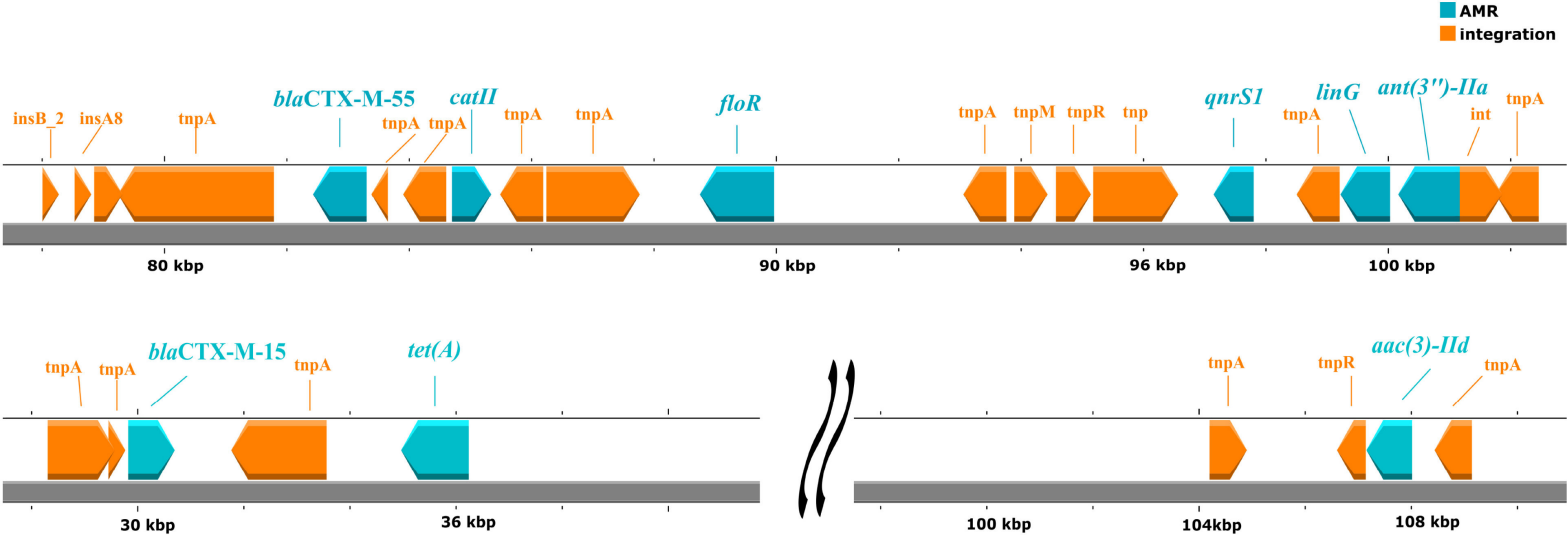
Genetic organization of antimicrobial resistance (AMR) regions in plasmids of *Salmonella* isolates GGN-BG-22 and GGN-BG-25. Genetic contexts of AMR genes with adjacent mobile genetic element-associated genes are shown for GGN-BG-22 (upper panel) and GGN-BG-25 (lower panel). AMR genes are indicated in cyan, whereas insertion sequences, transposases, and integrases are indicated in orange. These resistance regions were associated with mobile genetic contexts in both plasmids. The *ant(3*″)*-IIa*-like aminoglycoside resistance gene in GGN-BG-22 was annotated as a pseudogene.

Selected chromosomal and plasmid-associated virulence features of the two representative isolates are summarized in Tables S9 and S10, whereas representative plasmid structures and AMR-associated regions are shown in Fig. 2 and 3. Comparative circular chromosome maps are provided in Fig. S3. GGN-BG-22 harbored a putative SGI-4-like chromosomal region containing clustered *sil* and *pco* metal tolerance genes together with multiple ICE-associated mobility genes. This region included the *sil* locus (*silE-silS-silR-silC-silB-silA-silP*) and the *pco* locus (*pcoA-pcoB-pcoC-pcoD-pcoR-pcoS*), along with an integrase domain-containing protein, a tyrosine-type recombinase/integrase, a TraI domain-containing protein, TraD, a TraG N-terminal domain-containing protein, a PilL N-terminal domain-containing protein, and additional conjugation-associated proteins. Collectively, these features support the interpretation of this locus as an SGI-4-associated ICE-like chromosomal region rather than an isolated metal tolerance locus.

The complete plasmid sequence of GGN-BG-22 (CP196702.1) was a 107,357-bp circular plasmid carrying an IncFIB replicon [IncFIB(AP001918); 97.65% identity, 682/682 bp]. This plasmid harbored multiple resistance-associated genes, including *bla*_CTX-M-55_, *qnrS1*, *floR*, *catA2*, and *lnu(F*), whereas an *ant(3*″)-*IIa*-like aminoglycoside resistance gene was present as a pseudogene. In addition, the plasmid retained substantial transfer-associated gene content, with most *tra* genes and several *trb* genes identified, together with *finO*. Multiple mobile genetic elements were also present, including IS26 copies, numerous transposase-related genes, and several integrase-associated genes, including an *intI1* pseudogene.

The characteristic virulence loci of classical *S*. Typhimurium virulence plasmids, including *spv*, *pef*, and *rck*, were not identified.

In the chromosome of GGN-BG-25, *sopE* was located at 1,969,915–1,970,637 within a Gifsy_2-like region that was classified as intact by PHASTEST and spanned 1,953,154–1,984,776. This region contained multiple phage- and recombination-associated genes, including a tyrosine-type recombinase/integrase, excisionase, bacteriophage antitermination protein Q, phage holin, lysozyme, lysis protein, phage minor tail protein L, phage tail tip fiber protein, and a phage terminase large subunit family protein, along with several pseudogenized recombinase- and tail/fiber-related genes. *sopE2*, however, was located outside this predicted region.

The GGN-BG-25 plasmid carried IncFIB(S) and IncFII(S) replicons and harbored *bla*_CTX-M-15_, *tet(A*), and *aac(3)-IId*. It also carried plasmid-associated virulence determinants, including *pef*, *spv*, *rck*, and *mig-5*.

### SNP-based phylogeny

**Fig 4:**
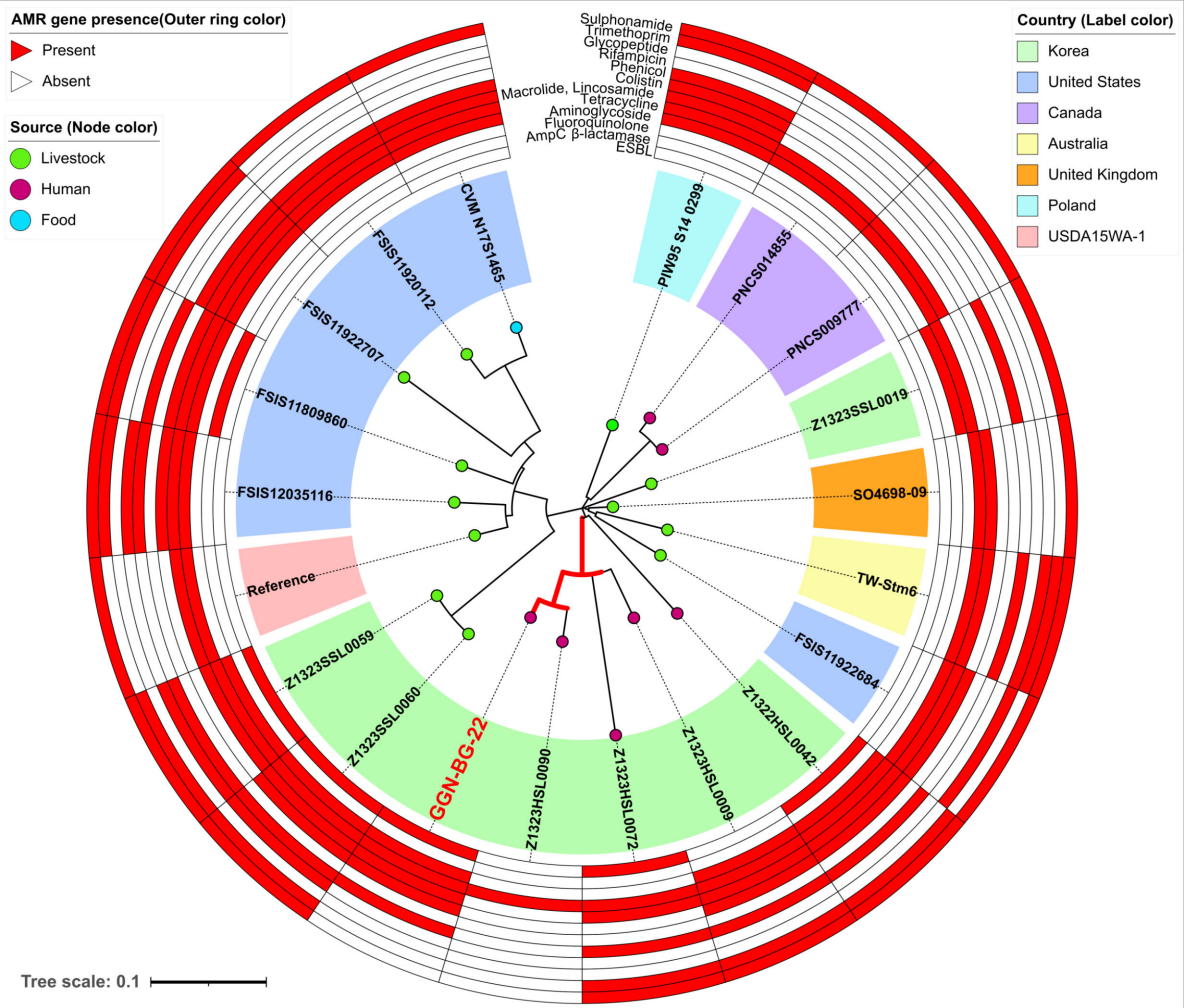
Single-nucleotide polymorphism (SNP)-based phylogenetic tree of *S*. I 4,[5],12:i:- isolate GGN-BG-22 within a selected ST34 comparative genomic data set. The maximum-likelihood tree was generated from core genome SNPs using USDA15WA-1 as the reference strain. Publicly available comparator genomes were selected from the corresponding sequence type. Isolates are annotated by country or reference-strain status, source, and antimicrobial resistance (AMR) gene presence using label color, node color, and outer-ring annotations, respectively. This tree provides comparative genomic context for GGN-BG-22 within the available comparison set. Metadata for comparator genomes included in the analysis are summarized in Table S3. The scale bar indicates nucleotide substitutions per site.

**Fig 5:**
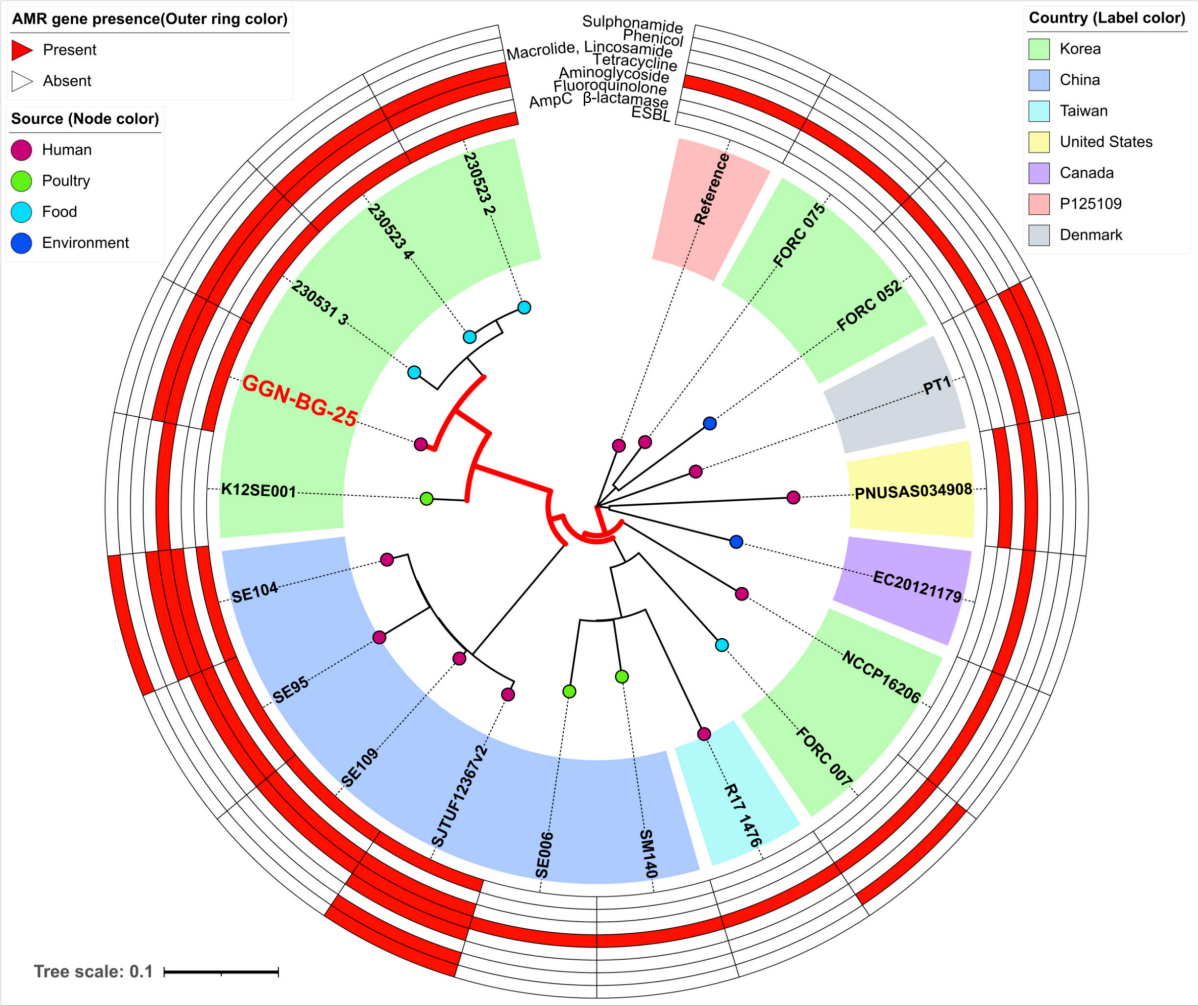
Single-nucleotide polymorphism (SNP)-based phylogenetic tree of *S*. Enteritidis isolate GGN-BG-25 within a selected ST11 comparative genomic data set. The maximum-likelihood tree was generated from core genome SNPs using P125109 as the reference strain. Publicly available comparator genomes were selected from the corresponding sequence type. Isolates are annotated by country or reference-strain status, source, and antimicrobial resistance (AMR) gene presence using label color, node color, and outer-ring annotations, respectively. This tree provides comparative genomic context for GGN-BG-25 within the available comparison set. Metadata for comparator genomes included in the analysis are summarized in Table S4. The scale bar indicates nucleotide substitutions per site.

Core genome SNP analysis was performed using 445 high-quality SNPs for the *S*. I 4,[5],12:i:- data set and 463 for the *S*. Enteritidis data set (Fig. 4 and 5, respectively). Using selected publicly available genomes, GGN-BG-22 was positioned within a broader ST34 background and showed closer relatedness to Korean isolates (Fig. 4). GGN-BG-25 clustered most closely with several Korean ST11 isolates, including food-associated isolates (Fig. 5). The accompanying AMR annotations facilitated comparison of resistance-associated features across the analyzed genomes. Metadata and associated antimicrobial resistance gene profiles for the genomes included in these analyses are summarized in Tables S3 and S4.

## DISCUSSION

In this study, we characterized 320 clinical *Salmonella enterica* isolates obtained from patients with diarrhea in northern Gyeonggi Province, Korea, between 2015 and 2022. Among the isolates selected for molecular typing, the major multidrug-resistant lineages included *S*. I 4,[5],12:i:- ST34 and *S*. Enteritidis ST11, suggesting that these lineages represented important resistant groups in this regional clinical collection. Although these lineages have been reported in other settings, the present findings should be interpreted primarily in the context of regional surveillance rather than broader global dissemination (38–41).

Resistance was highest to ampicillin, tetracycline, and nalidixic acid, and 26.6% of isolates exhibited MDR. Extended-spectrum cephalosporin resistance was mainly associated with *bla*_CTX-M_ and *bla*_DHA_, whereas plasmid-mediated quinolone resistance determinants, such as *qnrS* and *qnrB*, were also detected. Clear serotype-specific differences were also observed, with *S*. Enteritidis showing significantly higher resistance to nalidixic acid and colistin and *S*. I 4,[5],12:i:- showing higher resistance to tetracycline, chloramphenicol, and trimethoprim-sulfamethoxazole. The high resistance observed in *S*. I 4,[5],12:i:- is consistent with previous reports describing this monophasic variant as a lineage frequently associated with multidrug resistance (42).

The coexistence of β-lactamase genes, plasmid-mediated quinolone resistance determinants, and QRDR alterations highlights the complexity of resistance evolution in these isolates (4–6, 17–23). In particular, the frequent detection of mutations in *gyrA* corresponding to the predicted D87N substitution, together with other QRDR variants and PMQR genes, suggests an increased risk of reduced fluoroquinolone susceptibility (43). In addition, isolates carrying *bla*_CTX-M-65_ remained susceptible to CAZ, indicating that CTX-M allele identity should be interpreted together with phenotypic susceptibility profiles rather than assuming uniform cephalosporin resistance patterns (44, 45).

PFGE suggested genetic relatedness within major resistant serotypes, while MLST assigned selected resistant isolates to major sequence types. WGS further provided higher-resolution genomic context for two representative isolates. Together, these complementary approaches linked population-level surveillance findings with isolate-level genomic features.

GGN-BG-22 carried a putative SGI-4-like chromosomal region containing clustered *sil* and *pco* metal tolerance genes together with multiple ICE-associated mobility genes, consistent with genomic features previously described in monophasic ST34-associated mobile chromosomal regions (46). Although the canonical full-length SGI-4 structure could not be unequivocally resolved, the coexistence of clustered metal tolerance genes and mobility-associated loci supports interpretation of this region as an SGI-4-associated ICE-like chromosomal locus.

The GGN-BG-22 plasmid was genetically distinct from the classical *S*. Typhimurium virulence plasmid pSLT and is better interpreted as a multidrug resistance-associated IncFIB plasmid (47, 48). The absence of *spv*, *pef*, and *rck* is inconsistent with a classical virulence plasmid architecture, whereas the presence of *bla*_CTX-M-55_, *qnrS1*, *floR*, *catA2*, and *lnu(F*) indicates accumulation of clinically relevant resistance determinants (47, 48). The retention of most *tra* genes together with several *trb* genes and *finO* further suggests preservation of a substantial transfer-associated backbone, although conjugation was not experimentally confirmed.

In GGN-BG-25, the chromosomal context of *sopE* suggests that this effector is embedded within a phage-derived or prophage-like chromosomal segment rather than a typical core genomic region (49, 50). This interpretation is supported by the PHASTEST prediction of a Gifsy_2-like region and by the presence of recombination-, lysis-, and tail/fiber-associated genes flanking *sopE*. However, because canonical structural and boundary-associated features of a fully intact prophage were not clearly identified, a conservative interpretation of this locus is warranted.

The GGN-BG-25 plasmid also carried IncFIB(S) and IncFII(S) replicons together with *bla*_CTX-M-15_, *tet(A*), and *aac(3)-IId*. The coexistence of these resistance determinants with plasmid-associated virulence genes, including *pef*, *spv*, *rck*, and *mig-5*, suggests that this ST11-associated plasmid may provide genetic potential for both antimicrobial resistance and virulence-associated traits (47).

The SNP-based phylogenetic analyses shown in Fig. 4 and 5 provided comparative genomic context for the two representative isolates within selected publicly available comparison data sets. Within this framework, GGN-BG-22 was positioned within a broader ST34 background and showed closer relatedness to Korean comparator isolates. GGN-BG-25 clustered most closely with several Korean ST11 comparator isolates, including food-associated strains. The accompanying AMR annotations further supported interpretation of relatedness in the context of shared or distinct resistance-associated profiles. These analyses should be interpreted as comparative placement within the available comparison set rather than as a comprehensive reconstruction of global population structure.

A few limitations of this study should be noted. First, all isolates were collected from a single geographic region, although the predominant serotypes and resistance profiles were broadly consistent with previous clinical surveillance data from Korea. Second, WGS was performed for only two representative isolates, which limits broader genomic inference. Third, detailed clinical metadata were not available for all patients. Future studies including a larger number of complete genomes from diverse serotypes and settings will be needed to strengthen genomic epidemiological interpretation.

In conclusion, among molecularly typed MDR *Salmonella* isolates from northern Gyeonggi Province, ST34 and ST11 were major lineages associated with diverse β-lactamase genes, quinolone resistance determinants, and mobile genetic elements. The combined use of PFGE, MLST, and focused comparative genomics showed that these molecularly characterized lineages were associated with distinct resistance- and virulence-related genomic features. These findings support the continued need for molecular surveillance and antimicrobial stewardship to monitor and control MDR *Salmonella* in clinical and public health settings.

## Data Availability

The complete genome sequences generated in this study have been deposited in NCBI GenBank under accession numbers CP196701–CP196702 (GGN-BG-22) and CP196402–CP196403 (GGN-BG-25). Raw sequencing reads are available in the NCBI Sequence Read Archive (SRA) under BioProject accession number PRJNA1290791.

## SUPPLEMENTAL MATERIAL

Tables S1 to S10; Fig. S1 to S3 spectrum.03400-25-s0001.docx Tables S1: Primers used for β-lactamase genes and QRDR-associated genes in *Salmonella* isolates. Tables S2: Primers used for PCR amplification of PMQR genes in *Salmonella* isolates. Tables S3: AMR gene profiles of selected ST34 *S. enterica* serovar I 4,[5],12:i:- comparators for GGN-BG-22 phylogeny. Tables S4: AMR gene profiles of selected ST11 *S. Enteritidis* comparators for GGN-BG-25 phylogeny. Tables S5: Summary of major multidrug resistance profiles among 85 MDR *Salmonella* isolates. Tables S6: QRDR mutation patterns among 110 *Salmonella* isolates. Tables S7: PMQR gene patterns detected among 20 PMQR-positive *Salmonella* isolates. Tables S8: STs of 38 *Salmonella* isolates selected for MLST analysis. Tables S9: Selected chromosomally encoded virulence-associated genes in GGN-BG-22 and GGN-BG-25. Tables S10: Selected plasmid-associated virulence-related genes in GGN-BG-22 and GGN-BG-25. Fig. S1: Pulsed-field gel electrophoresis dendrogram of *S. Bareilly*, *S. Typhimurium*, and *S. Virchow* isolates. Fig. S2: Phylogenetic tree of multilocus sequence typing housekeeping gene sequences from 35 selected *Salmonella* isolates. Fig. S3: Comparative circular chromosome maps of S. I 4,[5],12:i:- isolate GGN-BG-22 and *S. Enteritidis* isolate GGN-BG-25.

ASM does not own the copyrights to Supplemental Material that may be linked to, or accessed through, an article. The authors have granted ASM a non-exclusive, world-wide license to publish the Supplemental Material files. Please contact the corresponding author directly for reuse.
