# Supplementary material for: Antimicrobial resistance surveillance and focused comparative genomics of clinical Salmonella isolates from patients with diarrhea in northern Gyeonggi Province, Korea
Source: Microbiol Spectr. 2026 Jul 14;14(8):e03400-25. doi: 10.1128/spectrum.03400-25 (PMC13436367; doi:10.1128/spectrum.03400-25)
Supplement: Supplementary file 1 [file spectrum.03400-25-s0001.docx]

# Supplemental Material

# Manuscript ID: Spectrum03400-25

# Antimicrobial Resistance Surveillance and Focused Comparative Genomics of Clinical *Salmonella* Isolates from Patients with Diarrhea in Northern Gyeonggi Province, Korea

# Supplementary Tables

**Supplementary Table S1.** Primers used for PCR amplification of β-lactamase genes and QRDR-associated genes in *Salmonella* isolates

| **Target gene** | **Primer** | **Sequence (5′–3′)** | **Product size (bp)** | **Reference** |
| --- | --- | --- | --- | --- |
| *bla*_CTX-M_ (Group I) | CTX-M I-F | TCCAGAATAAGGAATCCCATGG | 621 | 17 |
|  | CTX-M I-R | TGCTTTACCCAGCGTCAGAT |  |  |
| *bla*_CTX-M_ (Group II) | CTX-M II-F | ACCGCCGATAATTCGCAGAT | 588 | 17 |
|  | CTX-M II-R | GATATCGTTGGTGGTGCCATAA |  |  |
| *bla*_CTX-M_ (Group IV) | CTX-M IV-F | GACAAAGAGAGTGCAACGGATG | 501 | 17 |
|  | CTX-M IV-R | TCAGTGCGATCCAGACGAAA |  |  |
| *bla*_TEM_ | TEM-F | AGTGCTGCCATAACCATGAGTG | 431 | 17 |
|  | TEM-R | CTGACTCCCCGTCGTGTAGATA |  |  |
| *bla*_OXA_ | OXA-F | ATTATCTACAGCAGCGCCAGTG | 296 | 17 |
|  | OXA-R | TGCATCCACGTCTTTGGTG |  |  |
| *bla*_SHV_ | SHV-F | GATGAACGCTTTCCCATGATG | 214 | 17 |
|  | SHV-R | CGCTGTTATCGCTCATGGTAA |  |  |
| *bla*_CMY_ | CMY II-F | AGCGATCCGGTCACGAAATA | 695 | 17 |
|  | CMY II-R | CCCGTTTTATGCACCCATGA |  |  |
| *bla*_DHA_ | DHA-F | GTGGTGGACAGCACCATTAAA | 314 | 17 |
|  | DHA-R | CCTGCGGTATAGGTAGCCAGAT |  |  |
| *gyrA* | GYRA-F | TGTCCGAGATGGCCTGAAGC | 470 | 18 |
|  | GYRA-R | CGTTGATGACTTCCGTCAG |  |  |
| *gyrB* | GYRB-F | AAGCGCGATGGCAAAGAAG | 1257 | 18 |
|  | GYRB-R | AACGGTCTGCTCATCAGAAAGG |  |  |
| *parC* | PARC-F | ATGAGCGATATGGCAGAGCG | 413 | 18 |
|  | PARC-R | TGACCGAGTTCGCTTAACAG |  |  |
| *parE* | PARE-F | GACCGAGCTGTTCCTTGTGG | 492 | 18 |
|  | PARE-R | GCGTAACTGCATCGGGTTCA |  |  |

Primers for *bla*_CMY_ targeted the CMY-II group.

**Supplementary Table S2.** Primers used for PCR amplification of plasmid-mediated quinolone resistance genes in *Salmonella* isolates

| **Target gene** | **Primer** | **Sequence (5′–3′)** | **Product size (bp)** | **Reference** |
| --- | --- | --- | --- | --- |
| *qnrA* | qnrA-F | ATTTCTCACGCCAGGATTTG | 468 | 19 |
|  | qnrA-R | TGCCAGGCACAGATCTTGAC |  |  |
| *qnrB* | qnrB-F | GATCGTGAAAGCCAGAAAGG | 469 | 20 |
|  | qnrB-R | ACGATGCCTGGTAGTTGTCC |  |  |
| *qnrC* | qnrC-F | GGGTTGTACATTTATTGAATCG | 307 | 23 |
|  | qnrC-R | CACCTACCCATTTATTTTCA |  |  |
| *qnrD* | qnrD-F | CGAGATCAATTTACGGGGAATA | 582 | 22 |
|  | qnrD-R | AACAAGCTGAAGCGCCTG |  |  |
| *qnrS* | qnrS-F | ACGACATTCGTCAACTGCAA | 417 | 20 |
|  | qnrS-R | TAAATTGGCACCCTGTAGGC |  |  |
| *qepA* | qepA-F | AACTGCTTGAGCCCGTAGAT | 596 | 19 |
|  | qepA-R | GTCTACGCCATGGACCTCAC |  |  |
| *oqxA* | oqxA-F | CTCGGCGCGATGATGCT | 392 | 21 |
|  | oqxA-R | CCACTCTTCACGGGAGACGA |  |  |
| *oqxB* | oqxB-F | TTCTCCCCCGGCGGGAAGTAC | 512 | 21 |
|  | oqxB-R | CTCGGCCATTTTGGCGCGTA |  |  |
| *aac(6*′*)-Ib* | aac-F | TATGAGTGGCTAAATCGAT | 482 | 20 |
|  | aac-R | CCCGCTTTCTCGTAGCA |  |  |

The *aac(6′)-Ib* PCR target was sequenced to identify the *aac(6′)-Ib-cr* variant.

**Supplementary Table S3.** Antimicrobial resistance gene profiles of selected ST34 *S*. I 4,[5],12:i:- comparator genomes used for SNP-based phylogenetic analysis of GGN-BG-22

| **Isolate** | **Country** | **Source** | **ESBL** | **AmpC** | **FQ** | **AG** | **TET** | **ML** | **COL** |
| --- | --- | --- | --- | --- | --- | --- | --- | --- | --- |
| CVM_N17S1465 | USA | Food | **-** | **-** | **-** | *aph(3')-Ia* | *tet(B)* | *msr(E)* | *mcr-9.1* |
| FSIS11809860 | USA | Livestock | **-** | *bla*_CMY-2_ | **-** | *aph(3')-Ia* | *tet(B)* | *msr(E)* | *-* |
| FSIS11920112 | USA | Livestock | **-** | **-** | **-** | *aph(3')-Ia* | *tet(B)* | *msr(E)* | *mcr-9.1* |
| FSIS11922684 | USA | Livestock | **-** | **-** | **-** | *aac(3)-IId* | *tet(B)* | *msr(E)* | *-* |
| FSIS11922707 | USA | Livestock | **-** | **-** | **-** | *aph(3')-Ia* | *tet(B)* | *msr(E)* | *mcr-9.1* |
| FSIS12035116 | USA | Livestock | **-** | **-** | **-** | *aph(3')-Ia* | *tet(B)* | *lnu(F)* | *-* |
| GGN-BG-22 | Korea | Human | *bla*_CTX-M-55_ | **-** | *qnrS1* | *aac(6')-Iaa* | *tet(B)* | *lnu(F)* | *-* |
| PIW95_S14_0299 | Poland | Livestock | *-* | **-** | *-* | *aph(3')-Ia* | *tet(B)* | *mef(B)* | *mcr-9.1* |
| PNCS009777 | Canada | Human | *-* | **-** | *-* | *aac(6')-Iaa* | *tet(B)* | **-** | **-** |
| PNCS014855 | Canada | Human | *-* | **-** | *-* | *aac(6')-Iaa* | *tet(B)* | **-** | **-** |
| SO4698-09 | UK | Livestock | *-* | **-** | *-* | *aac(6')-Iaa* | *tet(B)* | **-** | **-** |
| TW-Stm6 | Australia | Livestock | *-* | **-** | *-* | *aph(3')-IIa* | *tet(A)* | **-** | **-** |
| USDA15WA-1^†^ | USA | Livestock | *-* | **-** | *-* | *aac(6')-Iaa* | *tet(B)* | **-** | **-** |
| Z1322HSL0042 | Korea | Human | *bla*_CTX-M-65_ | **-** | *qnrS1* | *aph(4)-Ia* | *tet(B)* | *mef(B)* | **-** |
| Z1323HSL0009 | Korea | Human | *-* | **-** | *qnrS1* | *aph(4)-Ia* | *tet(A)* | *mef(B)* | **-** |
| Z1323HSL0072 | Korea | Human | *bla*_CTX-M-65_ | **-** | *-* | *aph(3')-Ia* | *tet(A)* | **-** | **-** |
| Z1323HSL0090 | Korea | Human | *-* | **-** | *-* | *aac(6')-Iaa* | **-** | **-** | **-** |
| Z1323SSL0019 | Korea | Livestock | *-* | **-** | *qnrS1* | *aadA1* | **-** | **-** | **-** |
| Z1323SSL0059 | Korea | Livestock | *bla*_CTX-M-65_ | **-** | *qnrS1* | *aph(4)-Ia* | *tet(A)* | **-** | **-** |
| Z1323SSL0060 | Korea | Livestock | *bla*_CTX-M-65_ | **-** | *qnrS1* | *aph(4)-Ia* | *tet(A)* | *lnu(F)* | **-** |

**Abbreviations:** ESBL, extended-spectrum β-lactamase; AmpC, AmpC β-lactamase; FQ, fluoroquinolones; AG, aminoglycosides; TET, tetracyclines; ML, macrolide- and lincosamide-associated resistance genes; COL, colistin.Only selected antimicrobial resistance gene classes are shown. A dash (-) indicates that no resistance gene was detected in the corresponding antimicrobial class. ^†^ USDA15WA-1 was used as the reference strain for the ST34 SNP-based phylogenetic analysis.

**Supplementary Table S4.** Antimicrobial resistance gene profiles of selected ST11 *S*. Enteritidis comparator genomes used for SNP-based phylogenetic analysis of GGN-BG-25

| **Isolate** | **Country** | **Source** | **ESBL** | **AmpC** | **FQ** | **AG** | **TET** | **ML** | **PHEN** |
| --- | --- | --- | --- | --- | --- | --- | --- | --- | --- |
| 230523_2 | Korea | Food | *bla*_CTX-M-15_ | - | - | *aac(3)-IId* | *tet(A)* | - | - |
| 230523_4 | Korea | Food | *bla*_CTX-M-15_ | - | - | *aac(3)-IId* | *tet(A)* | - | - |
| 230531_3 | Korea | Food | *bla*_CTX-M-15_ | - | - | *aac(3)-IId* | *tet(A)* | - | - |
| EC20121179 | Canada | Environment | - | - | - | *aac(6')-Iaa* | - | - | - |
| FORC_007 | Korea | Food | - | - | - | *aac(6')-Iaa* | - | - | *catA2* |
| FORC_052 | Korea | Environment | - | - | - | *aac(6')-Iaa* | - | - | - |
| FORC_075 | Korea | Human | - | - | - | *aac(6')-Iaa* | - | - | - |
| GGN-BG-25 | Korea | Human | *bla*_CTX-M-15_ | - | - | *aac(3)-IId* | *tet(A)* | - | - |
| K12SE001 | Korea | Poultry | - | - | - | *aac(6')-Iaa* | - | - | - |
| NCCP16206 | Korea | Human | - | - | - | *aac(6')-Iaa* | - | - | - |
| P125109^†^ | UK | Human | - | - | - | *aac(6')-Iaa* | - | - | - |
| PNUSAS034908 | USA | Human | - | *bla*_CMY-2_ | - | *aac(6')-Iaa* | - | - | - |
| PT1 | Denmark | Human | - | - | - | *aac(6')-Iaa* | - | - | - |
| R17_1476 | Taiwan | Human | - | - | - | *aac(6')-Iaa* | - | *mph(A)* | *floR* |
| SE006 | China | Poultry | - | - | - | *aac(6')-Iaa* | - | - | - |
| SE95 | China | Human | *bla*_CTX-M-55_ | - | *oqxAB* | *aph(3')-IIa* | - | - | - |
| SE104 | China | Human | *bla*_CTX-M-55_ | - | *oqxAB* | *aph(3')-Ia* | *tet(A)* | - | - |
| SE109 | China | Human | *bla*_CTX-M-55_ | - | *oqxAB* | *aph(3')-IIa* | - | - | - |
| SJTUF12367v2 | China | Human | *bla*_CTX-M-55_ | - | *oqxAB* | *aph(3')-IIa* | *tet(A)* | - | *floR* |
| SM140 | China | Poultry | - | - | - | *aac(6')-Iaa* | - | - | - |

**Abbreviations:** ESBL, extended-spectrum β-lactamase; AmpC, AmpC β-lactamase; FQ, fluoroquinolones; AG, aminoglycosides; TET, tetracyclines; ML, macrolide-associated resistance genes; PHEN, phenicol.

Only selected antimicrobial resistance gene classes are shown. A dash (-) indicates that no resistance gene was detected in the corresponding antimicrobial class. ^†^ P125109 was used as the reference strain for the ST11 SNP-based phylogenetic analysis.

**Supplementary Table S5.** Summary of major multidrug resistance profiles among 85 MDR *Salmonella* isolates

| **MDR category** | **No. of MDR isolates, n (%)** | **Major resistance patterns** | **n (%) among 85 MDR isolates** |
| --- | --- | --- | --- |
| 5-class MDR | 4 (4.7) | NAL, COL, CTX, CRO, CAZ, AMP, TET, GEN CTX, CRO, AMP, TET, CHL, GEN, SXT | 2 (2.4) |
|  |  |  | 2 (2.4) |
| 4-class MDR | 43 (50.6) | NAL, COL, AMP, TET AMP, TET, CHL, SXT NAL, CTX, CRO, CAZ, AMP, TET, GEN | 18 (21.2) |
|  |  |  | 13 (15.3) |
|  |  |  | 5 (5.9) |
| 3-class MDR | 38 (44.7) | NAL, AMP, TET NAL, COL, AMP CTX, CRO, CAZ, AMP, TET, CHL | 19 (22.4) |
|  |  |  | 11 (12.9) |
|  |  |  | 4 (4.7) |

**Abbreviations:** MDR, multidrug resistance; NAL, nalidixic acid; COL, colistin; CTX, cefotaxime; CRO, ceftriaxone; CAZ, ceftazidime; AMP, ampicillin; TET, tetracycline; CHL, chloramphenicol; GEN, gentamicin; SXT, trimethoprim-sulfamethoxazole.

Values are presented as number (%) of MDR isolates. Only the major resistance patterns within each MDR category are shown. Percentages were calculated among 85 MDR isolates.

**Supplementary Table S6.** QRDR mutation patterns among 110 *Salmonella* isolates with reduced susceptibility to nalidixic acid or ciprofloxacin

| ***gyrA*** | ***gyrB*** | ***parC*** | ***parE*** | **No. of isolates** |
| --- | --- | --- | --- | --- |
| D87N | — | — | — | 67 |
| D87N | — | T57S | — | 2 |
| D87Y | — | — | — | 9 |
| D87Y | — | T57S | — | 2 |
| D87G | — | — | — | 5 |
| S83F | — | — | — | 2 |
| S83Y | — | — | — | 1 |
| S83Y | V252I | T57S | — | 1 |
| S83Y | — | T57S | — | 1 |
| — | — | T57S | — | 6 |
| — | — | — | S493Y | 1 |
| — | — | — | — | 13 |

Predicted amino acid substitutions are shown using one-letter amino acid notation. Some isolates carried multiple substitutions across different QRDR-associated genes. —, no substitution detected in the corresponding QRDR-associated gene.

**Supplementary Table S7.** Plasmid-mediated quinolone resistance gene patterns detected among 20 PMQR-positive Salmonella isolates

| **PMQR gene pattern** | **No. of isolates** |
| --- | --- |
| *qnrS1* | 13 |
| *qnrB4* | 1 |
| *qnrB19* | 4 |
| *qnrB19 + qnrS1* | 1 |
| *oqxA + oqxB + aac(6′)-Ib-cr5* | 1 |
| Total | 20 |

Values represent PMQR-positive isolates. Among the screened PMQR targets, *qnrA*, *qnrC*, *qnrD*, and *qepA* were not detected. The *aac(6′)-Ib* PCR target was sequenced to identify the *aac(6′)-Ib-cr* variant.

**Supplementary Table S8.** Sequence types (STs) of 38 *Salmonella* isolates selected for MLST analysis

| **No** | **Sample ID** | **Serotype** | **Allele Profile*** | **ST** | **Year** |
| --- | --- | --- | --- | --- | --- |
| 1 | GGN-BG-10 | *S*. I 4,[5],12:i:- | 10-19-12-9-5-9-2 | 34 | 2017 |
| 2 | GGN-BG-104 | *S*. I 4,[5],12:i:- | 10-19-12-9-5-9-2 | 34 | 2018 |
| 3 | GGN-BG-106 | *S*. I 4,[5],12:i:- | 10-19-12-9-5-9-2 | 34 | 2018 |
| 4 | GGN-BG-46 | *S*. I 4,[5],12:i:- | 10-19-12-9-5-9-2 | 34 | 2019 |
| 5 | GGN-BG-26 | *S*. I 4,[5],12:i:- | 10-19-12-9-5-9-2 | 34 | 2021 |
| 6 | GGN-BG-27 | *S*. I 4,[5],12:i:- | 10-19-12-9-5-9-2 | 34 | 2021 |
| 7 | GGN-BG-28 | *S*. I 4,[5],12:i:- | 10-19-12-9-5-9-2 | 34 | 2021 |
| 8 | GGN-BG-29 | *S*. I 4,[5],12:i:- | 10-19-12-9-5-9-2 | 34 | 2021 |
| 9 | GGN-BG-14 | *S*. I 4,[5],12:i:- | 10-19-12-9-5-9-2 | 34 | 2022 |
| 10 | GGN-BG-15 | *S*. I 4,[5],12:i:- | 10-19-12-9-5-9-2 | 34 | 2022 |
| 11 | GGN-BG-16 | *S*. I 4,[5],12:i:- | 10-19-12-9-5-9-2 | 34 | 2022 |
| 12 | GGN-BG-17 | *S*. I 4,[5],12:i:- | 10-19-12-9-5-9-2 | 34 | 2022 |
| 13 | GGN-BG-22 | *S*. I 4,[5],12:i:- | 10-19-12-9-5-9-2 | 34 | 2022 |
| 14 | GGN-BG-67 | *S*. I 4,[5],12:i:- | 10-19-12-9-5-9-2 | 34 | 2022 |
| 15 | GGN-BG-83 | *S*. I 4,[5],12:i:- | 10-19-12-9-5-9-2 | 34 | 2022 |
| 16 | GGN-BG-19 | *S*. I 4,[5],12:i:- | 10-19-363-9-5-9-2 | 2379 | 2022 |
| 17 | GGN-BG-9 | *S*. Enteritidis | 5-2-3-7-6-6-11 | 11 | 2017 |
| 18 | GGN-BG-24 | *S*. Enteritidis | 5-2-3-7-6-6-11 | 11 | 2021 |
| 19 | GGN-BG-25 | *S*. Enteritidis | 5-2-3-7-6-6-11 | 11 | 2021 |
| 20 | GGN-BG-30 | *S*. Enteritidis | 5-2-3-7-6-6-11 | 11 | 2021 |
| 21 | GGN-BG-31 | *S*. Enteritidis | 5-2-3-7-6-6-11 | 11 | 2021 |
| 22 | GGN-BG-12 | *S*. Enteritidis | 5-2-3-7-6-6-11 | 11 | 2022 |
| 23 | GGN-BG-35 | *S*. Bareilly | 81-69-36-12-68-12-17 | 203 | 2017 |
| 24 | GGN-BG-108 | *S*. Bareilly | 81-69-36-12-68-12-17 | 203 | 2017 |
| 25 | GGN-BG-109 | *S*. Bareilly | 81-69-36-12-68-12-17 | 203 | 2017 |
| 26 | GGN-BG-115 | *S*. Bareilly | 81-69-36-12-68-12-17 | 203 | 2021 |
| 27 | GGN-BG-80 | *S*. Bareilly | 81-69-36-12-68-12-17 | 203 | 2022 |
| 28 | GGN-BG-20 | *S.* Typhimurium | 120-7-12-9-5-9-2 | 323 | 2022 |
| 29 | GGN-BG-21 | *S.* Typhimurium | 120-7-12-9-5-9-2 | 323 | 2022 |
| 30 | GGN-BG-6 | *S.* Typhimurium | 10-7-12-9-5-9-2 | 19 | 2019 |
| 31 | GGN-BG-72 | *S.* Typhimurium | 10-7-1048-9-5-9-2 | 8316 | 2022 |
| 32 | GGN-BG-90 | *S.* Typhimurium | 10-7-12-†-5-9-† | Unassigned | 2021 |
| 33 | GGN-BG-36 | *S*. Virchow | 6-7-10-10-8-10-14 | 16 | 2015 |
| 34 | GGN-BG-37 | *S*. Virchow | 6-7-10-10-8-10-14 | 16 | 2015 |
| 35 | GGN-BG-69 | *S*. Virchow | 75-67-10-17-15-9-14 | 1750 | 2022 |
| 36 | GGN-BG-7 | *S*. Infantis | 17-18-22-17-5-21-19 | 32 | 2019 |
| 37 | GGN-BG-32 | *S*. Thompson | 14-13-18-12-14-18-1 | 26 | 2020 |
| 38 | GGN-BG-1 | *S*. Hadar | 2-5-6-7-5-7-12 | 33 | 2022 |

* Allele profile order: *aroC-dnaN-hemD-hisD-purE-sucA-thrA*. † The novel alleles detected in *hisD* and thrA were not assigned official allele numbers in the PubMLST database.

**Supplementary Table S9.** Selected chromosomally encoded virulence-associated genes in GGN-BG-22 and GGN-BG-25

| **Category** | **Locus/factor** | **GGN-BG-22** | **GGN-BG-25** |
| --- | --- | --- | --- |
| Fimbrial adherence | Peg | ND | *pegA, pegB, pegC, pegD* |
|  | Sef | ND | *sefC* |
|  | Stc | *stcA, stcB, stcC, stcD* | ND |
|  | Ste | ND | *steA, steB, steC, steD, steE, steF* |
|  | Stf | *stfB* | ND |
|  | Stj | *stjB, stjC* | ND |
| Secretion system | T3SS-1 translocated effectors | ND | *sopE* |
|  | T3SS-2 translocated effectors | *gogB* | ND |

**Abbreviations:** ND, not detected; T3SS, type III secretion system.

**Supplementary Table S10.** Selected plasmid-associated virulence-related genes in GGN-BG-22 and GGN-BG-25

| **Category** | **Locus/factor** | **GGN-BG-22 plasmid** | **GGN-BG-25 plasmid** |
| --- | --- | --- | --- |
| Fimbrial adherence | Pef | ND | *pefA, pefB, pefC, pefD* |
| Macrophage survival | Mig-5 | ND | *mig-5* |
| Secretion system | T3SS-2 translocated effectors | ND | *spvC, spvD* |
| Toxin | SpvB | ND | *spvB* |
| Serum resistance | Rck | ND | *rck* |
|  | TraT | *traT* | ND |

**Abbreviations:** ND, not detected; T3SS, type III secretion system.

# Supplementary Figures

**Supplementary Figure S1.** Pulsed-field gel electrophoresis dendrogram of S. Bareilly, S. Typhimurium, and S. Virchow isolates showing resistance to third-generation cephalosporins and/or reduced susceptibility to nalidixic acid or ciprofloxacin. Isolates are clustered by serotype, with accompanying metadata indicating resistance genes, QRDR mutations, antimicrobial susceptibility profiles (R, resistant; I, intermediate; S, susceptible), and year of isolation. Extended-spectrum β-lactamase-related genes and cephalosporin resistance are highlighted in red, while quinolone resistance-related genes, QRDR mutations, and quinolone/fluoroquinolone non-susceptibility are shown in blue.

**Supplementary Figure S2.** Neighbor-joining phylogenetic tree based on concatenated multilocus sequence typing housekeeping gene sequences from 35 selected *Salmonella* isolates representing five major serotypes collected in northern Gyeonggi Province, Korea. Isolates are grouped by sequence type (ST) and serotype, with metadata showing isolate ID, resistance determinants, antimicrobial susceptibility profiles (R, resistant; I, intermediate; S, susceptible), and year of isolation. β-lactamase-related genes and cephalosporin resistance are highlighted in red, while quinolone resistance-related genes, QRDR mutations, and quinolone/fluoroquinolone non-susceptibility are shown in blue. To improve readability, serotypes are color-coded: *S.* I 4,[5],12:i:- (green), *S.* Typhimurium (magenta), *S.* Bareilly (light blue), *S.* Enteritidis (orange), and *S.* Virchow (black).

**Supplementary Figure S3.** Comparative circular chromosome maps of *S*. I 4,[5],12:i:- isolate GGN-BG-22 and *S*. Enteritidis isolate GGN-BG-25. The maps display coding sequences, antimicrobial resistance (AMR)-associated genes, mobile genetic element-associated genes, regulatory genes, virulence-associated genes, and GC skew. AMR-associated genes are shown in blue, mobile genetic element-associated genes including transposases, integrases, and insertion sequences are shown in red, regulatory genes are shown in orange, and virulence-associated genes are shown in cyan. Selected chromosomal AMR-associated features, including efflux pump-associated genes and quinolone resistance-related mutations, are labeled.
